# Hei-Gu-Teng Zhuifenghuoluo Granule Modulates IL-12 Signal Pathway to Inhibit the Inflammatory Response in Rheumatoid Arthritis

**DOI:** 10.1155/2018/8474867

**Published:** 2018-05-29

**Authors:** Kang Zheng, Zexu Chen, Wen Sun, Bin Liu, Danping Fan, Qingqing Guo, Hui Luo, Jiawen Shen, Li Li, Xiaojuan He, Shuang Kou, Xiaoya Li, Guoming Pang, Hongchuan Zhao, Cheng Lu

**Affiliations:** ^1^Institute of Basic Research in Clinical Medicine, China Academy of Chinese Medical Sciences, Beijing 100700, China; ^2^Institute for Advancing Translational Medicine in Bone & Joint Diseases, School of Chinese Medicine, Hong Kong Baptist University, Kowloon Tong 00852, Hong Kong; ^3^College of the Second Clinical Medical, Guangzhou University of Chinese Medicine, Guangzhou 510000, China; ^4^College of Chemical and Biological Engineering, Yichun University, Yichun 336000, China; ^5^School of Life Science and Engineering, Southwest Jiaotong University, Chengdu 610000, China; ^6^Chinese Academy of Medical Sciences/Peking Union Medical College, Beijing 100193, China; ^7^Department of Diabetes, Kaifeng Hospital of TCM, Kaifeng 475000, China; ^8^Department of Gastroenterology, China-Japan Friendship Hospital, Beijing 100029, China

## Abstract

Rheumatoid arthritis (RA) is a type of chronic systemic inflammatory disease; it has a very complicated pathogenesis, and multiple pathological changes are implicated. Traditional Chinese medicine (TCM) like *Tripterygium wilfordii* Hook. F. or *Sinomenium acutum* (Thunb.) Rehd et Wils. has been extensively used for centuries in the treatment of arthritic diseases and been reported effective for relieving the severity of RA. Hei-Gu-Teng Zhuifenghuoluo granule (HGT) which contains *Periploca forrestii Schltr.*, *Sinomenium acutum* (Thunb.) Rehd et Wils., and *Lysimachia paridiformis* Franch. var. *stenophylla* Franch. was a representative natural rattan herb formula for the treatment of RA in China, but the mechanism has not been elucidated. This study aimed at exploring the mechanism of HGT on RA using the bioinformatics analysis with *in vivo* and *in vitro* experiment validation. The potential action mechanism was first investigated by bioinformatics analysis via Ingenuity Pathway Analysis (IPA) software. After that, we use experimental validation such as collagen-induced arthritis (CIA) mice model *in vivo* and U937 cell model *in vitro.* The bioinformatics results suggested that HGT may have anti-inflammatory characteristic on RA and IL-12 signaling pathway could be the potential key trigger. *In vivo* experiments demonstrated that HGT ameliorated the symptoms in CIA mice and decreased the production of inflammatory cytokines in both mice ankle joints and serum. Furthermore, HGT effectively inhibited the activation of IL-12R and STAT4 on IL-12 signaling pathway. *In vitro* experiments showed that HGT inhibited the production of IL-12R and STAT4 induced by IL-12 in lipopolysaccharide- (LPS-) stimulated U937 cells. Moreover, IL-12R knockdown was able to interfere with the inhibition effects of HGT on the production of these cytokines. Our results confirmed the anti-inflammatory property of HGT, which was attributed to its inhibition on IL-12 signaling pathway.

## 1. Introduction

Rheumatoid arthritis (RA) which manifests as persistent and progressive joint destruction is a common chronic autoimmune disease with inflammation which leads to pain, stiffness, and the final result functional disability [1, 2]. Cartilage destruction, bone fusions and erosion, chronic granulation, and scar tissue result in the joints including hands and feet [3]. Traditional Chinese medicine (TCM) including herbals and herbal formulas or extracts, such as Qingfuguanjieshu, Wu Tou Tang, and extracts of the herb *Tripterygium wilfordii* Hook. F., is valid for alleviating the inflammation severity of RA by demonstration [4, 5]. Some natural rattan herbals can provide good therapeutic effect in RA such as *Tripterygium wilfordii* Hook. F. [6] and *Sinomenium acutum* (Thunb.) Rehd et Wils. [7]; their active compounds or underlying mechanisms have always been a research trend as their main bioactive ingredient pharmacological efficacy may account for analgesic and anti-inflammatory activities [8].

Hei-Gu-Teng Zhuifenghuoluo granule (HGT) was an antiarthritic Chinese herbal formula in China [9], which is identified as the over-the-counter (OTC) drug for treatment of arthritis by the Chinese State Food and Drug Administration (CFDA), composed of *Periploca forrestii Schltr.*, *Sinomenium acutum* (Thunb.) Rehd et Wils., and *Lysimachia paridiformis* Franch. var. *stenophylla* Franch. Nowadays, investigators have not elucidated underlying molecular mechanism adequately.

Bioinformatics analysis technology, as a common way used for integrating multiple data, has become an essential way to help people understand organic life about biological relevant processes [10, 11]. Moreover, bioinformatics analysis method enables our required searching for the information of drugs, related genes, or proteins; constructs the interactional experimental system models; then makes underlying molecular interaction networks visualized. HGT consists of three herbals which were characterized by multicomponent and multitarget like other formulas. Based on abundantly existing databases, bioinformatics analysis can help better understand potential action mechanisms in the previous studies of our group [12, 13]. Therefore, in our study, we investigated the mechanism of HGT treatment on RA by bioinformatics analysis and *in vivo* and *in vitro* experimental validations.

## 2. Materials and Methods

### 2.1. Analysis of Molecular Networks and Signaling Pathways of HGT and RA

The human target proteins of HGT were found in PubChem platform (https://pubchem.ncbi.nlm.nih.gov/), and the key word was searched for in the PubChem Compound. The human target genes related with RA were found in the National Center for Biotechnology Information (NCBI) Gene database (http://www.ncbi.nlm.nih.gov/gene). “Rheumatoid arthritis” was used as a key word in Gene database searching. The obtained data were saved in an excel form for the next study [11, 13].

The human target protein and gene data acquired in the first step were imported into the IPA platform. The molecules being imputed to the IPA were termed “focus molecules.” IPA generated a set of networks based on different biofunctions. The molecules were showed as nodes, and the biological relationship between two nodes was showed as an edge (line). All edges were supported by at least one reference from a textbook, from the literature, or from canonical information stored in the IPKB. The nodes were showed with diverse shapes that represented the functional class of the gene product. The networks were sorted depending on the scores enumerated by IPA and represented the significance of the molecules for the network. The target protein networks of HGT and RA could be established. Some major information, such as top biological pathway network information, biological functions, canonical pathways, and other related bioanalytical information, was included. In order to study the mechanism of HGT on RA, the canonical pathway analysis in IPA was accomplished by using the compare module. In addition, IPA determined the significance of the association between the focus molecules and the canonical pathways using Fisher's exact test.

### 2.2. Cell Culture and Viability Assay

The human monocytic cell U937 (American Type Culture Collection, USA), as a classic cell model for RA research, was applied to the *in vitro* experiment [11]. They were placed in the fresh RPMI 1640 medium (GIBCO Chemical, USA) for 7-day cultivation at 37°C, accompanied by 10% FBS in it. For viability assay, the monocytic cells were planted at the surface of each well with an appropriate concentration of 1.0 × 10^5^ cells/mL in a 96-well plate, incubated with PMA (Sigma Aldrich Co., USA) at 37°C for stimulation. After 2-day stimulation, the cells were rinsed with PBS and then administrated with different concentrations of HGT (Beijing Handian Pharmaceutical Co., China) (0, 6.25, 12.5, 25, 50, 100, and 200 nM) for another 2 days. Then, 10 *μ*L of CCK-8 reagent (Dojindo Inc., Japan) was added and incubated for the last 3 h. The plate in an ELISA reader (Bio-Tek Instruments, USA) was placed, and the absorbance of each well at 450 nm was measured in the end.

### 2.3. Cell Administration

The U937 cells were planted at the surface of each well with a concentration of 1.0 × 10^6^ cells/mL in a 6-well plate and then treated with PMA for an incubation of 2 days at room temperature. After being rinsed with PBS for three times, the cells were harvested and then treated them or not with 100 ng/mL LPS (Sigma Aldrich Co., USA) for 2 h. Afterwards, the cells were administrated with a medium concentration (12.5 nM) of HGT for 2 days. At last, the supernatant and cells were collected for ELISA and western blot analysis, respectively.

### 2.4. Animals and Experimental Procedures

#### 2.4.1. Animals

Twenty-eight male DBA/1 mice (18–22 g) were kept in an appropriate environment on a light/dark cycle for 7 days. The animal experiments were complied with the Research Ethics Committee of the Institute of Basic Research in Clinical Medicine, China Academy of Chinese Medical Sciences, China. All the mice were supplied by the Laboratory Animal Center of the Academy of Military Medical Sciences, China.

#### 2.4.2. Induction of Arthritis Model

The mice collagen-induced arthritis (CIA) model was implemented on the basis of a previous study introduced by Smolen et al. [1]. Twenty-one mice were immunized through intradermal injection of 100 *μ*g bovine type II collagen (Chondrex, USA) into the base of the tail and meantime emulsified through supplementation with 100 *μ*L complete Freund's adjuvant (CFA) (Chondrex, USA). The day of the first immunization was determined on day 0 and the second immunization was on day 21; the animals received evaluation of severity degree two times every week. The degree was represented by the mean arthritic index on a 0–4 scale following the criterion below [2]: 0 = no swelling; 1 = slight swelling emerged on the foot and/or ankle; 2 = mild swelling emerged around the ankle and the tarsal; 3 = moderate swelling emerged around the ankle and the tarsal, accompanied by some erythema; and 4 = severe swelling emerged on the whole limb, accompanied by much erythema. Each limb was assessed, and the accumulated score was the final score. The mouse with a final score more than one score was judged to be a successful model.

#### 2.4.3. Administration

On day 35, the CIA mice were randomly divided into three groups: model group, HGT group, and MTX group and the other 7 healthy mice as a normal group. In the HGT group and MTX group, the mice received oral administration once a day. The mice in the normal group and model group received the same volume of saline. All the animals were sacrificed after 35-day administration. The serum and ankle joints were collected and stored at −80°C for the next studies.

#### 2.4.4. Histology and Immunohistochemistry

The ankle joints were first put into 4% paraformaldehyde for 48 h fixation, then immersed in 10% EDTA for 1 month decalcification, and at last embedded in paraffin for the next experiments. The tissues of each mouse were sectioned to 7 pieces, with a thickness of 6 *μ*m. For histology examination, the sections were stained with hematoxylin and eosin (HE). Synovial inflammation was evaluated with a three-point scale ranging from 0 to 3 as previous research described by Guo et al. [5].

For immunohistochemistry, the sections of the ankle joints were first deparaffinized and rehydrated and incubated with polyclonal rabbit anti-IL-12 (Abcam, UK) in a humidity cabinet overnight at 4°C. Then, a secondary peroxidase antibody was supplemented and reacted for 2 h. Finally, the sections were treated with DAB substrate solution. Each section was imaged under a light microscope (Carl Zeiss, Germany).

### 2.5. Enzyme-Linked Immunosorbent Assay (ELISA)

The levels of TNF-*α*, IL-1*β*, IL-4, IL-5, IL-6, IL-12, and IL-13 in cell culture supernatant and the levels of TNF-*α*, IL-1*β*, IL-6, and IL-12 in serum of the mice were measured by ELISA using unified ELISA kits (San Diego, USA) in the basis of the manufacturer's instructions.

### 2.6. Real-Time PCR

Frozen ankle tissues and monocytic cells were homogenized in nuclear lysis buffer (Trizol, Invitrogen, USA) for total RNA extraction, with a concentration of 5 × 10^6^ cells/mL. The lysate was collected and then centrifugated at room temperature with appropriate chloroform, isopropanol, and 75% ethanol, respectively. Afterwards, the precipitate was harvested, and some RNase-free water was added. The reverse transcriptions were performed using QuantiTect Reverse Transcription Kit (Qiagen K.K., Japan). The expressions of these cytokine mRNAs were quantified by defining the levels of each cytokine mRNA, and the mRNA levels of TNF-*α*, IL-1*β*, IL-6, and IL-12 were quantified by 7500 real-time PCR system (Applied Biosystems, USA). The data were figured out by the ΔΔCt algorithm and were unified based on the expression of GAPDH.

### 2.7. Western Blot Analysis

Cytosolic extracts of the U937 cells administrated with HGT and the ankle joints were prepared and homogenized or lysed in RIPA lysis buffer. The level of IL-12R*β*1 and STAT4 was quantified with anti-IL-12R*β*1 (Abcam, UK) and anti-STAT4 (Abcam, UK) in the cytosolic fraction from ankle joint tissues. Protein concentrations were quantified by the assay kit (Biotime Biotechnology, China) and were unified based on the expression of GAPDH. The gray value of each protein band was calculated by ImageJ.

### 2.8. Statistical Analysis

GraphPad Prism 7.0 and Student's *t*-test were used for statistical analysis. All experimental results were represented as the mean ± SD. *p* value less than 0.05 was judged as statistically significant.

## 3. Results

### 3.1. Results of Bioinformatics Analysis

**Figure 1:**
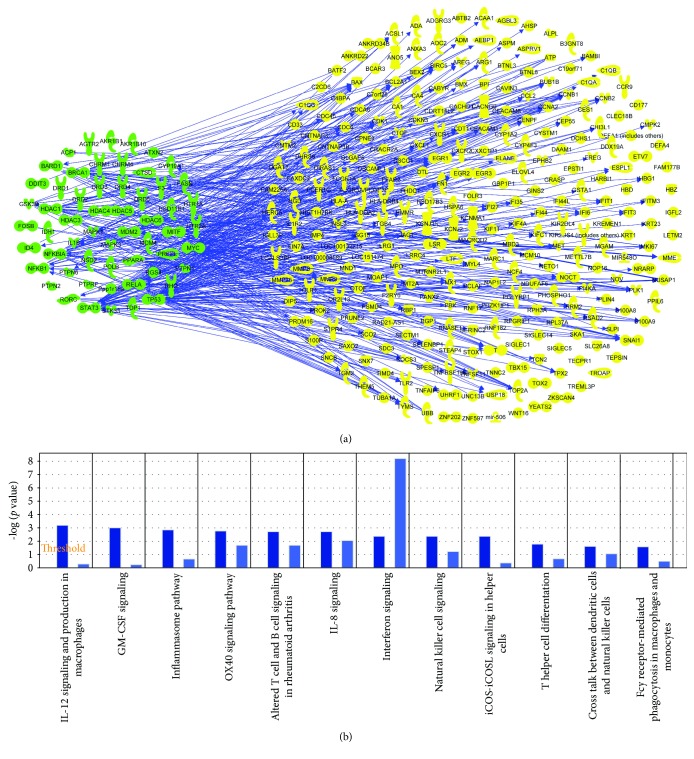
Bioinformatics analysis results. (a) The relationship between compound-related genes and RA disease-related genes; (b) shared signaling pathways between gene molecular networks related with rheumatoid arthritis (RA) and protein target molecular network of HGT performed using the Ingenuity Pathway Analysis (IPA) compare module. The signaling pathways of HGT were represented as dark blue, while the signaling pathways of RA were represented as light blue. The results showed that IL-12 signaling pathway was the top one shared signaling pathway.

Eight hundred and thirty-two genes related with RA were found from Gene database in NCBI. The top fifteen signaling pathways were focused on cellular immune response, cytokine signaling, humoral immune response, and intercellular and second-messenger signaling. Then, human target proteins of HGT were found from PubChem database. The details are shown in Table S1. After that, the molecular networks of HGT target proteins were obtained and shown in Figure 1(a), which included IL-12 signaling. We listed the top 12 shared signaling pathways of HGT and RA related to cell immune response (Figure 1(b)). Further comparative analysis showed that IL-12 signal pathway was measured to be the top one shared signaling pathway.

### 3.2. Effect of HGT on Paw Swelling in CIA Mice

**Figure 2:**
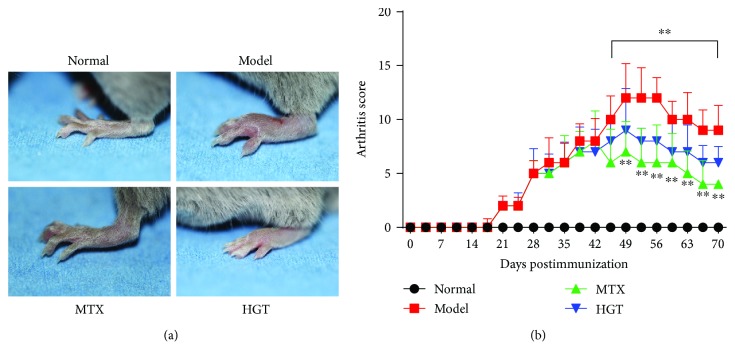
Effects of HGT on CIA mice. (a) The morphological characteristics of the representative of the CIA mice joint; (b) arthritic score in different days after the treatment of HGT; MTX represented the methotrexate group; HGT represented the HGT group. Date are represented as the mean ± SD (*n* = 7); ^∗∗^ *p* < 0.01, comparison with model group.

After CIA establishment, paw swelling in immunized mice on about day 42 reduced accordingly. As illustrated in Figure 2, treatment with HGT significantly suppressed inflammation. HGT could conspicuously inhibit paw swelling from after treatment (Figure 2(a)); the arthritis scores treated with HGT and MTX were decreased (Figure 2(b)).

### 3.3. Effect of HGT on Histopathological Changes in CIA Mice

**Figure 3:**
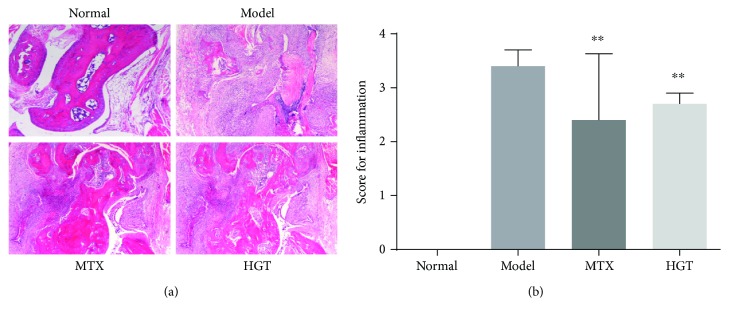
Effects of HGT on histopathological of CIA mice joints. (a) Histopathological characteristics of representative CIA mice joints; (b) inflammation score in the different group after treatment with HGT; MTX represented the methotrexate group, HGT represented the HGT group. Date are represented as the mean ± SD (*n* = 7); ^∗∗^ *p* < 0.01, comparison with the model group.

To evaluate inflammation induced by CIA, hematoxylin and eosin (H&E) staining was subsequently performed. As the results shown in Figure 3(a), there was no inflammatory cell infiltration in normal mice, but in CIA mice, there were clear slices exhibited. After treatment with HGT and MTX, inflammatory cell infiltration was noticeably decreased (Figure 3(b)).

### 3.4. Effect of HGT on Expression of Inflammatory Cytokines in Serum and CIA Mice Joints

**Figure 4:**
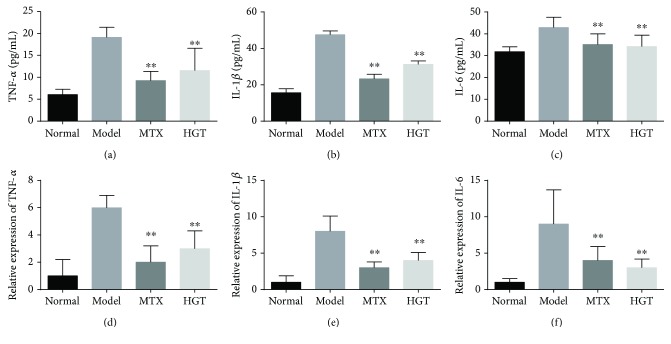
The effect of the expression of cytokines in mice. (a–c) IL-6, IL-1*β*, and TNF-*α* in the CIA mice with the treatment of HGT and also the mRNA expression in CIA mice joints with treatment. The data are represented as the mean ± SD (*n* = 7); ^∗∗^ *p* < 0.01, when compared with the model group.

As summarized in Figure 4, the expression of cytokines of TNF-*α* (Figure 4(a)), IL-1*β* (Figure 4(b)), and IL-6 (Figure 4(c)) in CIA mice serum was higher; HGT and MTX treatment both can significantly downregulate the levels. And then, the TNF-*α* mRNA (Figure 4(d)), IL-1*β* mRNA (Figure 4(e)), and IL-6 mRNA (Figure 4(f)) expression in CIA mice treated with HGT and MTX could also significantly decrease.

### 3.5. The Effect of the Expression of IL-12 and STAT4 in CIA Mice

**Figure 5:**
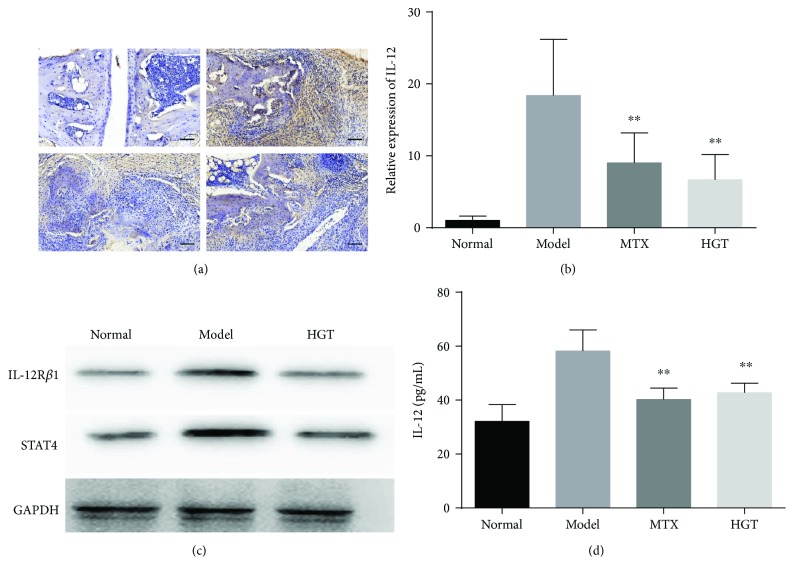
The effects of HGT on IL-12 and STAT4 expression in mice. (a) Immunohistochemistry of IL-12 in joints of mice with the treatment of HGT; (b) the levels of IL-12 mRNA in mouse joints after the treatment of HGT; (c) the expression of IL-12R*β*1 and STAT4 protein in CIA mice joints; (d) IL-12 in serum of CIA mice with the treatment of HGT. The data are represented as the mean ± SD (*n* = 7); ^∗∗^ *p* < 0.01, when compared with the model group.

To observe the effect of HGT on production of IL-12 and STAT4, we examined the expression of IL-12 and STAT4 in serum and ankle joints of CIA mice. The result indicated that HGT could decrease the production in both immunohistochemistry (Figure 5(a)) and serum (Figure 5(d)) and also mRNA expression (Figure 5(b)) in the ankle joints. Further, the protein levels of IL-12R*β*1 and STAT4 in mice ankle joints (Figure 5(c)) also markedly decreased after the treatment of HGT.

### 3.6. Effects of HGT on Inflammatory Cytokine Expression in Cell Model

**Figure 6:**
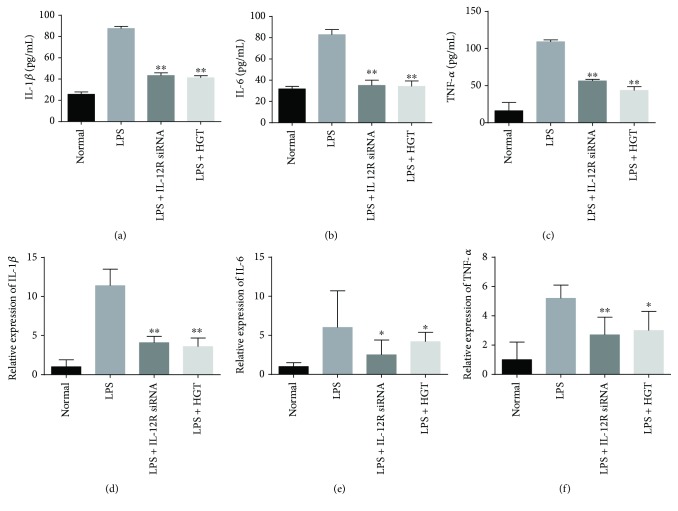
Effects of HGT on the expression of cytokines in U937 cells. (a–c) IL-6 levels, TNF-*α* levels, and IL-1*β* levels in LPS-induced U937 cells after the treatment of IL-12 siRNA and HGT; (d–f) IL-6, TNF-*α*, and IL-1*β* mRNA levels in LPS-induced U937 cells after treatment with IL-12 siRNA and HGT. The data are represented as the mean ± SD (*n* = 7); ^∗^ *p* < 0.05 and ^∗∗^ *p* < 0.01, when compared with the LPS group.

U937 cells were first used to stimulate with LPS, and then IL-1*β*, TNF-*α,* and IL-6 were measured. The results showed that the TNF-*α* levels, IL-1*β* levels, and IL-6 levels in the supernatant of U937 cells treated with HGT were inhibited both with ELISA measurement on secretion (Figures 6(a)–6(c)) and RT-PCR measurement of mRNA (Figures 6(d)–6(f)).

### 3.7. Effects of Expression of Cytokines of HGT after IL-12R*β*1 Knockdown

**Figure 7:**
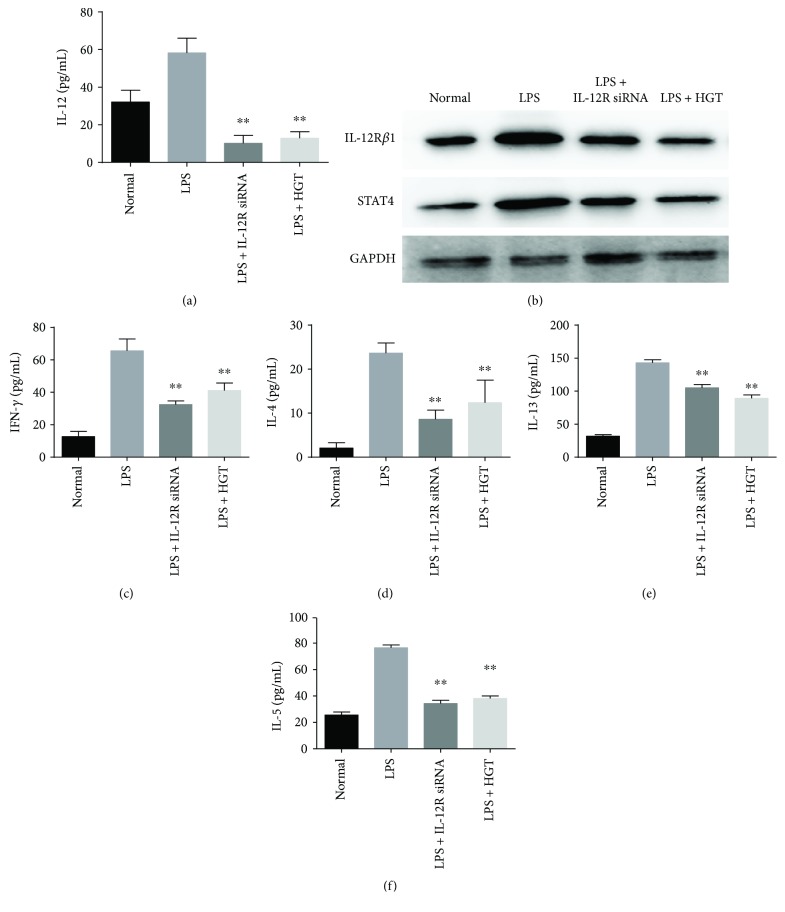
The effects of HGT on cytokine expression after IL-12R*β*1 knockdown. Firstly, the U937 cells were first treated with LPS and the other without LPS, and then all cells were transfected with IL-12R*β*1 siRNA. 48 h later, the U937 cells were then treated with HGT or without HGT for 48 h. (b) Western blot of the protein of IL-12R*β*1 and STAT4; (a, c, d, e, f) IL-12, IFN-*γ*, IL-4, IL-13, and IL-5 productions were determined by ELISA. The data are represented as the mean ± SD (*n* = 7); ^∗∗^ *p* < 0.01, when compared with the LPS group.

To determine the role in how the IL-12 signal pathway plays in the regulation of the effect HGT on LPS-induced inflammatory cytokine production, IL-12R*β*1 siRNA was used to knock down the expression of IL-12R*β*1. The results showed that IL-12 expression was significantly decreased in the U937 cells transfected with IL-12R*β*1 siRNA (Figure 7(a)). And also, the IL-12R*β*1 and STAT4 protein expression significantly decreased (Figure 7(b)). IL-12R*β*1 knockdown could decrease the levels of IFN-*γ* (Figure 7(c)) and upregulate the levels of IL-4 (Figure 7(d)), IL-5 (Figure 7(e)), and IL-13 (Figure 7(f)). And also, no significant difference was found between the cells transfected with IL-12R*β*1 siRNA and the cells transfected with IL-12R*β*1 siRNA in the presence of HGT.

### 3.8. Schematic Diagram Depicting How HGT Modulates the IL-12 Signaling Pathway to Inhibit the Inflammatory Response in Rheumatoid Arthritis

**Figure 8:**
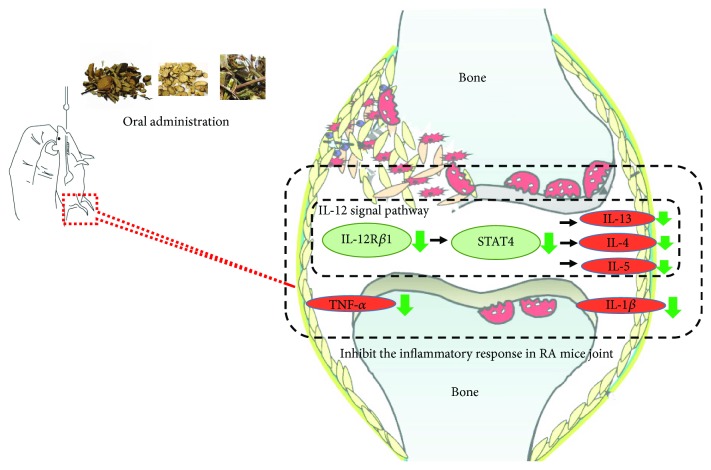
Schematic diagram depicting how HGT modulates the IL-12 signaling pathway to inhibit the inflammatory response in rheumatoid arthritis. By inhibiting IL-12R, HGT could inhibit STAT4 and then inhibit the inflammation, meanwhile, decreasing TNF-*α* and IFN-*γ*.

By inhibiting IL-12R, Figure 8 shows how HGT could inhibit STAT4 and then inhibit the inflammation, meanwhile, decreasing TNF-*α* and IFN-*γ*.

## 4. Discussion

Traditional Chinese medicine (TCM) has been used for arthritic diseases in China for hundreds of years extensively. Hei-Gu-Teng Zhuifenghuoluo granule (HGT) composed of *Periploca forrestii Schltr.*, *Sinomenium acutum* (Thunb.) Rehd et Wils., *and Lysimachia paridiformis* Franch. var. *stenophylla* Franch, was an antiarthritic Chinese herbal formula that was often used for treatment of joint pain and RA by Chinese doctors. In this herbal formula, certain bioactive chemicals such as sinomenine and *Periploca forrestii Schltr.* saponin have been previously identified [10, 11]. Sinomenine is a natural alkaloid isolated from the roots of *Sinomenium acutum* (Thunb.) Rehd et Wils and a variety of bioactivities have been reported such as anti-inflammatory and antirheumatic. Based on these effects, sinomenine has been widely applied in clinical treatment of RA in China. The dry root or whole vine of *Sinomenium acutum* (Thunb.) Rehd et Wils. was effective in clinical prescription for the treatment of rheumatoid diseases. Saponins are the characteristic components and also the main active ingredients of *Periploca forrestii Schltr.* saponin which was extracted from *Periploca forrestii Schltr.* that could prophylactically treat autoimmune arthritis by controlling the systemic autoimmune responses, local inflammation, and bone destruction of the joints [12].

Nevertheless, the progress of RA is very complicated, so more and more mechanisms should be elucidated. Bioinformatics is a method which could provide important avenues in organic life for biological relevant processes. Therefore, in order to investigate the potential mechanisms of HGT acting on RA, we employed an integrating network analysis of bioinformatics technology.

This bioinformatics analysis exposed that IL-12 signal pathway and IL-12 productions were related to RA and HGT, and this was consistent with some other previous studies [8, 13]. And the other analysis also indicated that IL-12 signal pathway was involved in the signaling pathway of cytokine and cellular immune in RA. Interleukin-12 (IL-12) is a heterodimeric cytokine which is produced primarily by antigen-presenting cells [14, 15]. It has many immunoregulatory effects on T cells and natural killer (NK) cells [16]. In this experiment, we verify that IL-12 levels in serum and CIA mice joints were increased, and HGT could also decrease the IL-12 expression both in mRNA and protein. Then, our experiment showed that LPS increased the IL-12 mRNA and IL-12 protein and the inflammation framework of U937 cells. As IL-12 is required for the promotion of Th1 development as well as IFN-*γ* expression [17, 18], IL-1*β*, IL-6, and TNF-*α* can be effected by STAT4. Our experiment showed both IL-12 knockdown and HGT could inhibit the production of IL-1*β*,TNF-*α*, and IL-6. And, the effects were retained after IL-12 knockdown, but there was no difference between cells transfected with IL-12 siRNA and cells transfected with IL-12 siRNA but treated with HGT simultaneously. IL-12R*β*1 plays an important role in IL-12 signaling pathway [19]. In this study, our bioinformatics analysis results showed that IL-12 signaling was involved in the inflammation regulation in HGT treatment of RA. STAT proteins like STAT1 or STAT4 are implicated in IL-12 signaling in T cells [20]. STAT4 is essential in mediating IL-12 function in T and NK cells [21, 22]. All major functions of IL-12 are abrogated in STAT4-deficient mice, including production of IFN-*γ* and NK cell cytotoxic activity [23, 24]. This suggests that regulating the expression of STAT4 may also be an important control point in modulating IL-12 function in NK and T cells [25, 26]. Therefore, our results suggested that HGT might modulate IL-12/STAT4 signal pathway in inhibiting inflammatory response. In sum, we confirmed the anti-inflammatory property of HGT, which was attributed to its inhibition on IL-12 signaling. Our finding also suggested that we can search the target of TCM and the possible mechanisms.

## 5. Conclusions

In conclusion, we confirmed the anti-inflammatory property of HGT, which was attributed to its inhibition on IL-12 signaling. Our finding also suggested that we can search the target of TCM and the possible mechanisms. Meanwhile, the consensus between the results analyzed by bioinformatics method and previous studies suggesting bioinformatics analysis method was reliable.

## Supplementary Materials

Supplementary Materials Table S1: human target proteins of HGT.
