# Supplementary material for: Hei-Gu-Teng Zhuifenghuoluo Granule Modulates IL-12 Signal Pathway to Inhibit the Inflammatory Response in Rheumatoid Arthritis
Source: J Immunol Res. 2018 May 29;2018:8474867. doi: 10.1155/2018/8474867 (PMC5996447; doi:10.1155/2018/8474867)
Supplement: Supplementary Materials — Table S1: human target proteins of HGT. [file 8474867.f1.docx]

Table S1 Human target proteins of HGT

| ID | Symbol | Entrez Gene Name |
| --- | --- | --- |
| 1709543 | ACP1 | acid phosphatase 1 |
| 1703214 | AGTR2 | angiotensin II receptor type 2 |
| 113596 | AKR1B1 | aldo-keto reductase family 1 member B |
| 322510010 | AKR1B10 | aldo-keto reductase family 1 member B10 |
| 171543895 | ATXN2 | ataxin 2 |
| 116241265 | BARD1 | BRCA1 associated RING domain 1 |
| 728984 | BRCA1 | BRCA1, DNA repair associated |
| 37622910 | CHRM1 | cholinergic receptor muscarinic 1 |
| 52426748 | CHRM4 | cholinergic receptor muscarinic 4 |
| 115717 | CTSD | cathepsin D |
| 117293 | CYP19A1 | cytochrome P450 family 19 subfamily A member 1 |
| 160707929 | DDIT3 | DNA damage inducible transcript 3 |
| 118228 | DRD1 | dopamine receptor D1 |
| 118229 | DRD1 | dopamine receptor D1 |
| 118206 | DRD2 | dopamine receptor D2 |
| 1169206 | DRD3 | dopamine receptor D3 |
| 118211 | DRD3 | dopamine receptor D3 |
| 1345939 | DRD4 | dopamine receptor D4 |
| 118214 | DRD5 | dopamine receptor D5 |
| 135666 | F3 | coagulation factor III, tissue factor |
| 1345958 | FASN | fatty acid synthase |
| 6679827 | FOSB | FosB proto-oncogene, AP-1 transcription factor subunit |
| 20455502 | GSK3B | glycogen synthase kinase 3 beta |
| 2498443 | HDAC1 | histone deacetylase 1 |
| 3334210 | HDAC3 | histone deacetylase 3 |
| 259016348 | HDAC4 | histone deacetylase 4 |
| 296434519 | HDAC5 | histone deacetylase 5 |
| 205371758 | HDAC6 | histone deacetylase 6 |
| 118569 | HSD11B1 | hydroxysteroid 11-beta dehydrogenase 1 |
| 231454 | HTR1A | 5-hydroxytryptamine receptor 1A |
| 543727 | HTR2A | 5-hydroxytryptamine receptor 2A |
| 881546 | ID4 | inhibitor of DNA binding 4, HLH protein |
| 49168486 | IDH1 | isocitrate dehydrogenase (NADP(+)) 1, cytosolic |
| 62203298 | IDH1 | isocitrate dehydrogenase (NADP(+)) 1, cytosolic |
| 10835145 | IL1B | interleukin 1 beta |
| 52001076 | MAPK1 | mitogen-activated protein kinase 1 |
| 52001483 | MAPK3 | mitogen-activated protein kinase 3 |
| 89993689 | MDM2 | MDM2 proto-oncogene |
| 89993689 | MDM2 | MDM2 proto-oncogene |
| 88702791 | MDM4 | MDM4, p53 regulator |
| 88702791 | MDM4 | MDM4, p53 regulator |
| 40807040 | MITF | melanogenesis associated transcription factor |
| 38156699 | MITF | melanogenesis associated transcription factor |
| 71774083 | MYC | MYC proto-oncogene, bHLH transcription factor |
| 34577122 | NFKB1 | nuclear factor kappa B subunit 1 |
| 61226507 | NFKBIA | NFKB inhibitor alpha |
| 109633019 | NSD2 | nuclear receptor binding SET domain protein 2 |
| 585064 | POLB | DNA polymerase beta |
| 3041727 | PPARA | peroxisome proliferator activated receptor alpha |
| 78486550 | Ppp1r15a | protein phosphatase 1, regulatory subunit 15A |
| 78486550 | Ppp1r15a | protein phosphatase 1, regulatory subunit 15A |
| 153217451 | PREPL | prolyl endopeptidase-like |
| 131467 | PTPN1 | protein tyrosine phosphatase, non-receptor type 1 |
| 229462762 | PTPN2 | protein tyrosine phosphatase, non-receptor type 2 |
| 131469 | PTPN6 | protein tyrosine phosphatase, non-receptor type 6 |
| 226709091 | PTPRF | protein tyrosine phosphatase, receptor type F |
| 417926 | RELA | RELA proto-oncogene, NF-kB subunit |
| 5032039 | RGS4 | regulator of G protein signaling 4 |
| 129204 | RHO | rhodopsin |
| 49066040 | RORC | RAR related orphan receptor C |
| 188536040 | RORC | RAR related orphan receptor C |
| 21618340 | STAT3 | signal transducer and activator of transcription 3 |
| 12830367 | STK33 | serine/threonine kinase 33 |
| 12830367 | STK33 | serine/threonine kinase 33 |
| 79154014 | TDP1 | tyrosyl-DNA phosphodiesterase 1 |
| 120407068 | TP53 | tumor protein p53 |
| 208342286 | TP53 | tumor protein p53 |
